# Enhanced multi-stress tolerance and glucose utilization of *Saccharomyces cerevisia*e by overexpression of the *SNF1* gene and varied beta isoform of Snf1 dominates in stresses

**DOI:** 10.1186/s12934-020-01391-4

**Published:** 2020-06-22

**Authors:** Lu Meng, Hui-Ling Liu, Xue Lin, Xiao-Ping Hu, Kun-Ru Teng, Si-Xin Liu

**Affiliations:** 1 College of Food Science and Engineering, Hainan University, Haikou, 570228 People’s Republic of China; 2 College of Science, Hainan University, Haikou, 570228 People’s Republic of China

**Keywords:** Stress tolerance, Snf1, Catalytic subunit, Regulatory subunit, *Saccharomyces cerevisiae*

## Abstract

**Background:**

The *Saccharomyces cerevisiae* Snf1 complex is a member of the AMP-activated protein kinase family and plays an important role in response to environmental stress. The α catalytic subunit Snf1 regulates the activity of the protein kinase, while the β regulatory subunits Sip1/Sip2/Gal83 specify substrate preferences and stress response capacities of Snf1. In this study, we aim to investigate the effects of *SNF1* overexpression on the cell tolerance and glucose consumption of *S. cerevisiae* in high glucose, ethanol, and heat stresses and to explore the valid Snf1 form in the light of β subunits in these stresses.

**Results:**

The results suggest that overexpression of *SNF1* is effective to improve cell resistance and glucose consumption of *S. cerevisiae* in high glucose, ethanol, and heat stresses, which might be related to the changed accumulation of fatty acids and amino acids and altered expression levels of genes involved in glucose transport and glycolysis. However, different form of β regulatory subunits dominated in stresses with regard to cell tolerance and glucose utilization. The Sip1 isoform was more necessary to the growth and glucose consumption in ethanol stress. The glucose uptake largely depended on the Sip2 isoform in high sugar and ethanol stresses. The Gal83 isoform only contributed inferior effect on the growth in ethanol stress. Therefore, redundancy and synergistic effect of β subunits might occur in high glucose, ethanol, and heat stresses, but each subunit showed specificity under various stresses.

**Conclusions:**

This study enriches the understanding of the function of Snf1 protein kinase and provides an insight to breed multi-stress tolerant yeast strains.

## Background

*Saccharomyces cerevisiae* is a typical eukaryotic model organism and widely used in fermentation industries. High-sugar fermentation is widely applied in various fermentation industry because of its high productivity, efficient equipment utilization, low energy consumption and production cost [1, 2]. However, *S. cerevisiae* cells inevitably suffer from high sugar osmotic pressure at the initial stage of fermentation. Simultaneously, increased temperature and toxic product such as ethanol stresses are usually generated accompanied with the fermentation [3, 4]. These stresses can destroy the cell structure and weaken the cell viability, affecting the quality and yield of final products. Therefore, improving the stress tolerance of *S. cerevisiae* cells is of great importance for promoting the development and application of high-sugar fermentation.

The *S. cerevisiae* Snf1 complex is a member of the AMP-activated protein kinase family and plays a critical role in a series of cellular activities, such as meiosis, aging, autophagy, and the responses to the limited glucose, nitrogen, and other environmental stresses [5, 6]. The Snf1 complex contains an α catalytic subunit Snf1, a γ regulatory subunit Snf4, and one of the three alternative β regulatory subunits Sip1, Sip2, or Gal83 [7]. The phosphorylation of Thr210 in the activation loop segment of α subunit Snf1 regulates the activity of the protein kinase [8]. The Snf4 subunit can counteract autoinhibition of the kinase through binding to the C-terminal regulatory region of Snf1 subunit [9]. The β subunits, which are required for the assemblage and intracellular localization of the protein kinase complex specify substrate preferences and stress response abilities of Snf1 [5, 10].

Previous studies showed that Snf1 phosphorylates repressor Mig1 in response to glucose effect relying on any of the three β isoforms [11]. The Sip1 form of Snf1 utilizes limited alternative carbon sources in Snf1-dependent regulation of alternative carbon sources metabolism; Gal83 is the most important isoform for the growth on non-fermentable carbon sources [12]. Deletion of the *SNF1* gene increased the sensitivities of yeast cells to Na^+^, Li^+^, hygromycin B, alkaline pH, oxidative, heat shock, and genotoxic stresses [13–16], suggesting that Snf1 served as a positive regulator to resist these stresses in yeast. On the contrary, Mizuno et al. [17] found the negative effect of *SNF1* mutation in endoplasmic reticulum stress. The Gal83 isoform of Snf1 was necessary in response to both alkaline pH and endoplasmic reticulum stresses [11, 17]. Sip2 exhibits potent functional activity equivalent to Gal83 in certain conditions and is implicated in aging [18, 19]. However, the functions of the *SNF1* gene of *S. cerevisiae* cells in high glucose and ethanol stresses and the form of β subunits participated in resistance to high glucose, ethanol, and heat stresses remain unclear.

In the present study, to investigate the effects of *SNF1* overexpression on the cell tolerance and glucose consumption of *S. cerevisiae* in high glucose, ethanol, and heat stresses and to explore the valid Snf1 form in the light of β subunits in coping with these stresses, firstly the genes *SNF1*, *SIP1*, *SIP2*, and *GAL83* were overexpressed in the *S. cerevisiae* strain AY3α alone to obtain the transformants α + S, α + SI1, α + SI2, and α + G83, respectively. Secondly, the cell viability and glucose consumption of the transformants and parental strain were tested in high glucose, ethanol, and heat stresses. Finally, the intracellular levels of cell protectants fatty acids and amino acids and the expression levels of genes involved in glucose transport and glycolysis of the transformants and parental strain were analyzed.

## Results

### Growth property

**Fig. 1:**
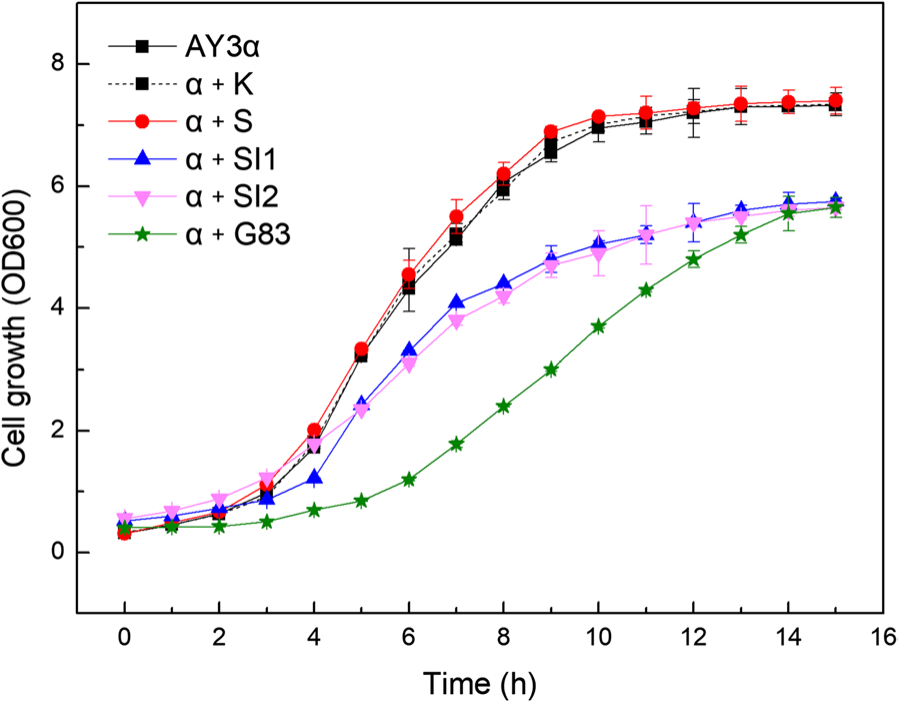
Growth curves of the strains. Growth curves were monitored by measuring the cell density (OD600) at appropriate time intervals

To investigate the influence of *SNF1*/*SIP1*/*SIP2*/*GAL83* overexpression on the general growth property of *S. cerevisiae*, the growth curves of the strains were measured in YEPD medium (Fig. 1). Compared to the parental strain AY3α, no significant differences of the growth characteristic were observed in the strain α + S. Nevertheless, the transformants α + SI1, α + SI2, and α + G83 exhibited poor proliferation in the same condition. The transformant α + K, which was used as a blank control to demonstrate any possible effects of an empty vector showed similar results to the parental strain.

These findings demonstrate that overexpression of *SNF1* did not influence the growth property of the *S. cerevisiae* strain used in the present study in a standard glucose growth condition (2% glucose), but any of the Snf1 complex dominated by only one β isoform served as a negative contributor.

### Stresses tolerance

**Fig. 2:**
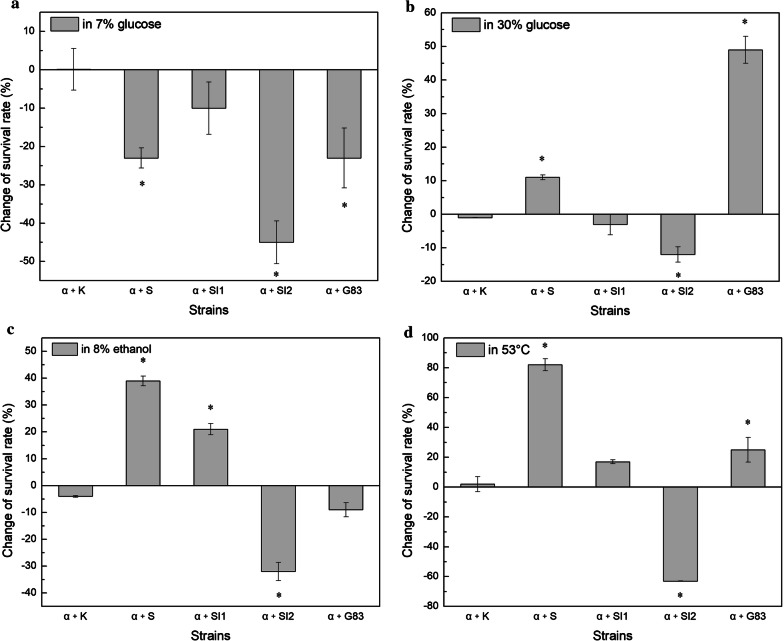
Analysis of stress tolerance. The cells were cultivated in **a** 70 g/L glucose at 30 °C, **b** 300 g/L glucose at 30 °C, **c** YEPD medium with 8% ethanol at 30 °C, and **d** YEPD medium at 53 °C. Change of the survival rate of the transformants was presented as the increase/decrease level of the relative cell viability relative to the base value of the parent strain AY3a. Significant difference of the transformants was confirmed by Student’s t-test (*P < 0.05)

To investigate the effect of *SNF1*/*SIP1*/*SIP2*/*GAL83* overexpression on the stress tolerance of *S. cerevisiae* cells, the relative cell viability and the change of the survival rate were calculated after treated with 30% glucose, 8% ethanol, and 53 °C (shown in Additional file 1 and Fig. 2, respectively). Compared to the parental strain AY3α, the cell survival rate increased by 11%, 39%, and 81% in the *SNF1* overexpression strain α + S after exposing to 30% glucose, 8% ethanol, and 53 °C, respectively. By contrast, the relative colony-forming number of the strain α + S was 23% lower than that of the parental strain AY3α in 7% glucose. The cell survival rate of the *SIP1* overexpression strain α + SI1 was 21% higher than that of the parental strain in 8% ethanol, but no obvious changes were found in 7% glucose, 30% glucose, and 53 °C. Compared to the parental strain, the *GAL83* overexpression strain α + G83 increased the cell survival rate by 49% and 24% in 30% glucose and 53 °C, respectively, whereas 23% decrease was observed in 7% glucose. The cell viability decreased by overexpression of *SIP2* in all of the conditions in this work. Similar behavior was found in the control transformant α + K to the parental strain.

These findings suggest that Snf1 played a positive role in regulation of high glucose, ethanol, and heat stresses response of yeast. However, the physical form of Snf1, which depended on β isoform was distinguished. A functional Snf1 mainly relied on the Gal83 isoform in high glucose and heat stresses, the Sip1 isoform functioned in high ethanol stress instead. Sip2-dependent form of Snf1 was negative to stress resistance of *S. cerevisiae* in the condition tested. The differences in structure and location of the three β subunits determine the specificity of Snf1-dependent regulation, and the three subunits show functional redundancy or independence rather than synergistic effect in stress and non-stress conditions [11, 12, 20].

### Glucose consumption

**Fig. 3:**
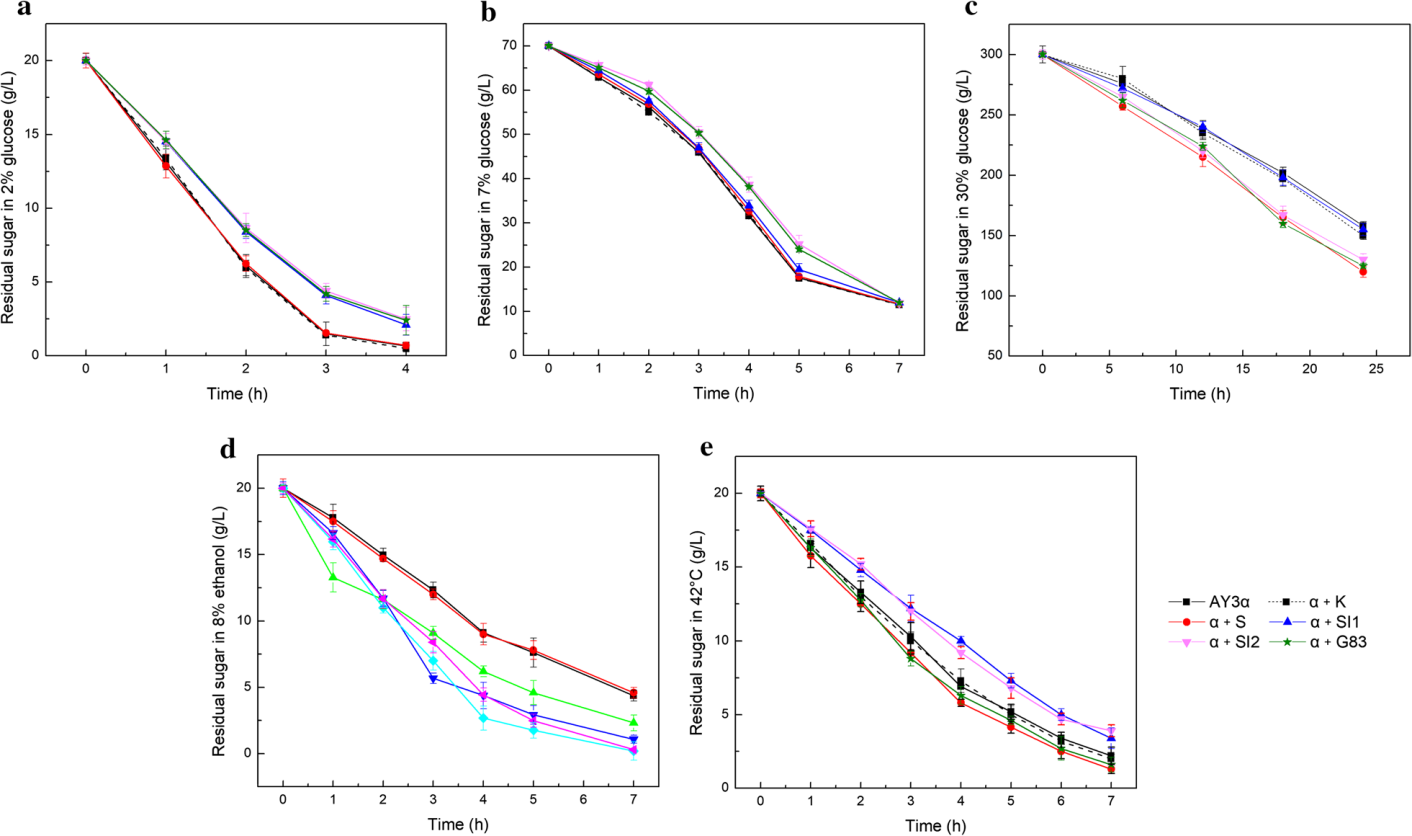
The residual glucose concentration of the strains. The consumption of glucose in **a** YEPD medium at 30 °C, **b** 70 g/L glucose at 30 °C, **c** 300 g/L glucose at 30 °C, **d** YEPD medium with 8% ethanol at 30 °C, and **e** YEPD medium at 42 °C

Considering Snf1 played a central role in adaption to glucose depletion without environmental stresses [6], the effect of *SNF1*/*SIP1*/*SIP2*/*GAL83* overexpression on the glucose utilization was investigated in the three stresses. Compared to the parental stain AY3α, the *SNF1* overexpression strain α + S increased the glucose utilization efficiency by 27%, 13%, and 5% in 30% glucose, 8% ethanol, and 42 °C, respectively, whereas no obvious difference was observed in 2% (Fig. 3). Meanwhile, the growth defect of the strain α + S did not affect the glucose consumption in 7% glucose. The glucose utilization of the *SIP1* overexpression strain α + SI1 was 21% higher than that of the parental strain in 8% ethanol, whereas those in 42 °C and 2% glucose were 7% decreases. No obvious changes of glucose utilization were found in the strain α + SI1 in 30% and 7% glucose. Compared to the parental stain, the *SIP2* overexpression strain α + SI2 displayed 20% and 27% increases of glucose utilization efficiency in 30% glucose and 8% ethanol, respectively, but 10% decrease was observed in 42 °C. Similar effects with 23% and 26% increases were observed in the *GAL83* overexpression strain α + G83 in 30% glucose and 8% ethanol, respectively, with slight change in 42 °C. The glucose consumption decreased in the strains α + SI2 and α + G83 in 2% and 7% glucose. The control transformant α + K exhibited no significant changes to the parental strain AY3α.

These results indicate that overexpression of *SNF1* was effective to glucose consumption of *S. cerevisiae* in high sugar, ethanol, and heat stresses. Nevertheless, the form of β isoforms conferring a functional Snf1 varied in different stresses. Non unique Sip1 isoform was necessary to the Snf1-dependent regulation in high glucose and synergistic effect might occur in the three β subunits. The Gal83 isoform was more important than Sip1 and Sip2 in response to heat stress. Snf1 carrying any of the three β isoforms contributed to the response to ethanol stress. The effect of *SNF1*/*SIP1*/*SIP2*/*GAL83* overexpression on the glucose utilization was corresponded to that on cell growth in 2% glucose, suggesting that three β isoforms jointly maintained glucose consumption in low glucose.

### Fatty acids and amino acids content

**Fig. 4:**
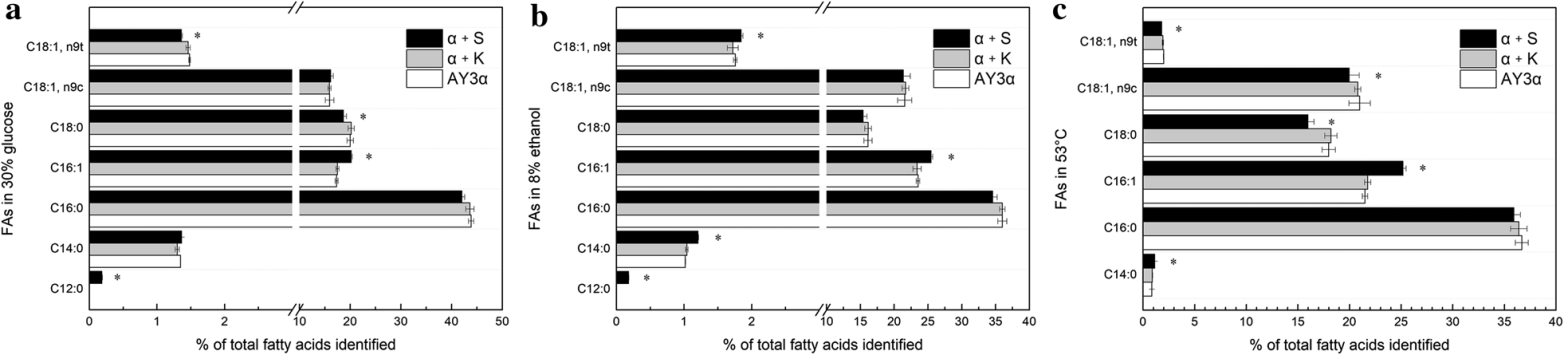
Relative percentage of fatty acids in the strains under different stresses. The relative content of fatty acids in the strains in **a** 300 g/L glucose at 30 °C, **b** YEPD medium with 8% ethanol at 30 °C, and **c** YEPD medium at 53 °C. The significant difference of the transformant α + S from the parental strain AY3α was confirmed by Student’s t-test (*P < 0.05)

The Snf1 protein kinase is a regulator of fatty acids and amino acids biosynthesis in low glucose [5, 21]. The composition and content of fatty acids and amino acids could affect the stress tolerance of yeast cells [22, 23]. Therefore, the levels of fatty acids and amino acids of the *SNF1* overexpression strain α + S were analyzed in high glucose, ethanol, and heat stresses. Similar results were found in the control transformant α + K to the parental strain. Compared to the parental strain, the proportion of C12:0, C16:1, C18:0, and C18:1n9t changed in the strain α + S in high glucose (Fig. 4a), and that in ethanol stress was C12:0, C14:0, C16:1, and C18:1n9t (Fig. 4b). The proportion of C18:1n9c was only evidently varied in heat stress (Fig. 4c). C12:0 was detected in the strain α + S in high glucose and ethanol condition, whereas it was not detected in the parental strain (Fig. 4a, 4b).

**Fig. 5:**
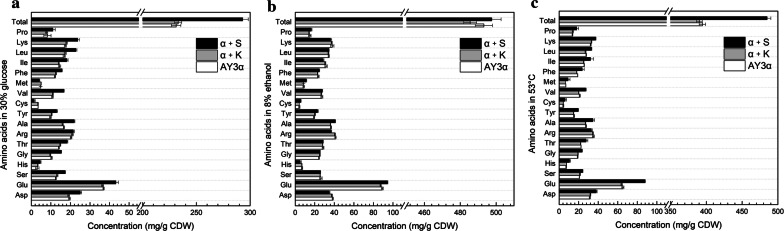
Amino acids content of the strains under different stresses. The content of amino acids in the strains in **a** 300 g/L glucose at 30 °C, **b** YEPD medium with 8% ethanol at 30 °C, and **c** YEPD medium at 53 °C. The contents of amino acids in the transformant α + S showed significant difference (confirmed by Student’s t-test, *P < 0.05) to that of the parental strain AY3α without that of Leu, Phe, Val, Thr, Gly, Ser, and total amino acids in 8% ethanol

For the accumulation of amino acids, compared to the parental strain, the total content of amino acids in the strain α + S was 26% and 25% higher than that in the parental strain in high glucose and heat stresses, respectively (Fig. 5a, c), but slight change was observed in ethanol stress (Fig. 5b). The addition of amino acids such as glutamic acid, lysine, arginine, and proline could facilitate cells to escape injury from stresses [24, 25]. Moreover, organisms could natively accumulate amino acids like glutamic acid and proline to avoid the lethal damage [26, 27]. Compared to the parental strain AY3α, the content of glutamic acid in the strain α + S increased by 17%,6%, and 34% in high glucose, ethanol, and heat stresses, respectively, and that for proline was 24%, 10%, and 119% increases. Interestingly, the proline content of the strains was corresponded with the results of stress tolerance in the three stresses, suggesting the importance of storage of proline in cell stress of yeast [27].

These results indicate that altering the expression of *SNF1* can modify the component and proportion of medium-chain/long-chain fatty acids, the proportion of saturated/unsaturated fatty acids, and the content of amino acids of *S. cerevisiae* in high glucose, ethanol, and heat stresses. Saturation degree and chain length of fatty acids are important factors that affect cell membrane fluidity. Increasing the proportion of unsaturated fatty acids and reducing the average length of fatty acids chain could increase the fluidity of cell membrane, thereby enhancing cell tolerance in stresses [28–31]. Amino acids could protect yeast cells from stress damage via maintaining the stability of proteins or membranes, lowering the *T*_*m*_ of DNA, inhibiting protein aggregation during refolding or folding, and scavenging reactive oxygen species [24, 27, 32].

### Expression level of genes involved in glucose transport and glycolysis

**Fig. 6:**
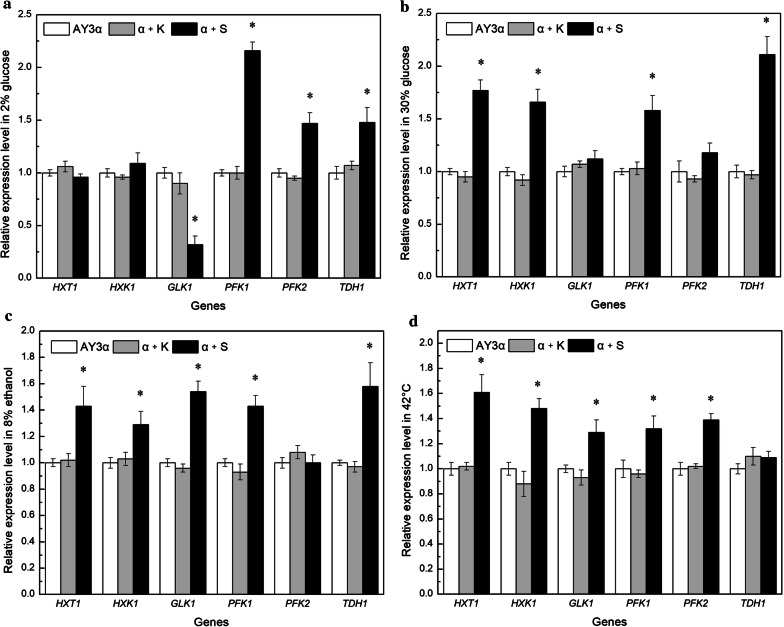
mRNA level of the genes involved in glucose transport and glycolysis. The cells were cultivated in **a** YEPD medium at 30 °C, **b** 300 g/L glucose at 30 °C, **c** YEPD medium with 8% ethanol at 30 °C, and **d** YEPD medium at 42 °C. The significant difference of the transformant α + S from the parental strain AY3α was confirmed by Student’s t-test (*P < 0.05)

Based on the results of glucose consumption in stresses, the mRNA levels of genes involved in glucose transport and glycolysis were analyzed to further investigate the effect of *SNF1* overexpression on glucose metabolism. The qRT-PCR results showed that the control strain α + K displayed no significant differences from the parental strain AY3α (Fig. 6). Compared to the parental strain, the levels of *HXT1* mRNA in the strain α + S increased by 77%, 43%, and 61% in high glucose, ethanol, and heat stresses, respectively, and that of *HXK1* was 66%, 29%, and 48% increases, respectively, but those in 2% glucose were unchanged. The transcription of *GLK1* in the strain α + S was up-regulated by 54% and 29% in ethanol and heat stresses, respectively, whereas that in 2% glucose was down-regulated by 68%, compared to the parental strain. The mRNA levels of *PFK1*, *PFK2*, and *TDH1* increased by 116%, 47%, and 48% in the strain α + S before stresses. Similar effect but with less increases of *PFK1* mRNA level were found in high glucose, ethanol, and heat stresses, and that for *PFK2* was uniquely observed in heat stress with 39% increase; more increases (111% and 58%, respectively) for *TDH1* were displayed in high glucose and ethanol stresses.

These results indicate that overexpression of *SNF1* had multi-effect on the expression of genes involved in glucose transport and glycolysis of *S. cerevisiae* in diverse conditions. *SNF1* overexpression up-regulated the expression of *GLK1*, *PFK1*, *PFK2*, and *TDH1* with no obvious effect on glucose uptake in 2% glucose, suggesting that mutation of *SNF1* exerted a transcriptional adaptation to the metabolic re-arrangement with altered expression of glycolytic genes [33]. Overexpression of *SNF1* enhanced the transcription of glucose transported and glycolytic genes to varying degrees in stresses. These findings were consistent with the results of Hu et al. [34], which reported that silencing the *SNF1* gene inhibited glycolysis of *Ganoderma lucidum* in heat stress. Enhanced glucose transportation and glycolysis flux could accelerate the decomposition of glucose and increase the substrate for amino acid synthesis in stresses.

## Discussion

Yeast strains capable of resisting stress are important to industrial production. Regulation of downstream intracellular effectors and midstream transcription factors are widely applied to improve cell stress [35–37]. Snf1, which is an upstream global regulatory factor is essential to control cell stress and survival. In the present study, we focused on the effect of overexpression of *SNF1*, which encodes the catalytic subunit of Snf1, on the resistance to high sugar, ethanol, and heat stresses of *S. cerevisiae*. Moreover, the form of β regulatory subunits dominated in the three stresses was investigated.

In this study, overexpression of *SNF1* improved the stress tolerance of *S. cerevisiae* in high sugar, ethanol, and heat stresses, which might be related to the changed accumulation of fatty acids and amino acids. The Snf1 protein kinase regulates the cellular processes not only through controlling the genomic transcription but also through directly influencing the activity of metabolic enzymes [5]. The function of *SNF1* involved in activating positive transcription factors or inhibiting negative transcription factors, which participate in the regulation of metabolically related genes for cell protectants, such as *MSN2*/*MSN4* in fatty acid and amino acid metabolism and *CAT8* in glycerol metabolism [38–40], might be activated in stresses. Thus, the yeast metabolome reshaped and more protectants accumulated for cell stress through overexpression of *SNF1*. Alternatively, the changed content of metabolite could be caused by the direct effect on the activity and stability of enzymes in *SNF1* overexpression, even by increasing the synthesis of precursor and cofactor of protectant because Snf1 regulates energy metabolism like AMPK in mammals [41]. However, deletion of *SNF1* was beneficial to attenuate the histone acetylation and promote the de novo synthesis of reduction equivalents, thereby increasing the accumulation of long-chain fatty acids in low glucose [33]. Enhancement of TCA activity and reduction of amino acids-dependent growth increased the accumulation of glutamic acid by deletion of *SNF1* in low glucose [33]. Snf1 inhibits the translation of the Gcn4 transcription factor and thus suppress the biosynthesis of amino acids and fatty acids in amino acid-rich and glucose-limiting conditions, respectively [5, 42]. These findings show that the role of Snf1 shifted and the different regulation programs were initiated in signals. The specific signal pathways and mechanisms of Snf1 in regulation of fatty acids and amino acids metabolism in glucose-rich and stress conditions need to be revealed in the future study.

Overexpression of *SNF1* negatively affected the growth of yeast in 7% glucose, suggesting different regulation mechanism of Snf1 for *S. cerevisiae* growth under non stress and stress conditions. This finding was corresponded with the results of Nicastro et al. [33], which reported that the slow-growth phenotype was lost in the *SNF1* mutant in 5% glucose. It is probably due to that excess *SNF1* inhibited the cell proliferation or accelerated the autophagy or destroyed the energy state in a 7% glucose-induced inhibitory state [43, 44]. Sip2/Gal83- but not Sip1-dependent form of Snf1 rendered inferior growth in 7% glucose, which differed from that in 2% glucose. This result suggests that glucose regulated the physical form and function of Snf1 complex [10, 34, 45]. The functions that β subunits exerted largely depended on the culture condition and genetic characteristic of strains. Although each β subunit alone is sufficient to grow in various conditions where Snf1 activity is required [38], the shared responsibility of β subunits might be essential to the growth of *S. cerevisiae* in the Snf1-dispensable condition such as 2% glucose.

In this study, overexpression of *SNF1* enhanced the glucose consumption of *S. cerevisiae* in high glucose, ethanol, and heat stresses. This could be attributed to the altered expression level of genes involved in glucose transport and glycolysis. The improvement of cell tolerance could increase glucose metabolism at certain levels as well by *SNF1* overexpression. However, the results of glucose utilization showed discrepancy to that of stress tolerance by β subunits overexpression in the three stresses tested, suggesting different mechanism of Snf1 in the regulation of growth and glucose metabolism of *S. cerevisiae* in response to stresses. Snf1 is regulated at the level of subcellular localization, which is directed by the β subunits [5, 46]. Signal molecules provide three β subunits with different location, and the β subunits possess specific subcellular localization pattern relying on their diverse N-terminal sequences [47]. The Sip1, Sip2, and Gal83 subunits enrich in the vacuolar, cytoplasm, and nucleus, respectively, in a glucose-depleted condition, but they are cytoplasmic when glucose is supplemented [5, 10]. Gal83 is enriched in the nucleus in response to alkaline pH but not salt stress [13]. The localization characteristic of β subunits could determine the response mechanism regulated by Snf1 in yeast. In addition, the structure of β subunits could affect the combination of substrate and generate altered regulation of various Snf1-dependent processes [11].

## Conclusions

**Fig. 7:**
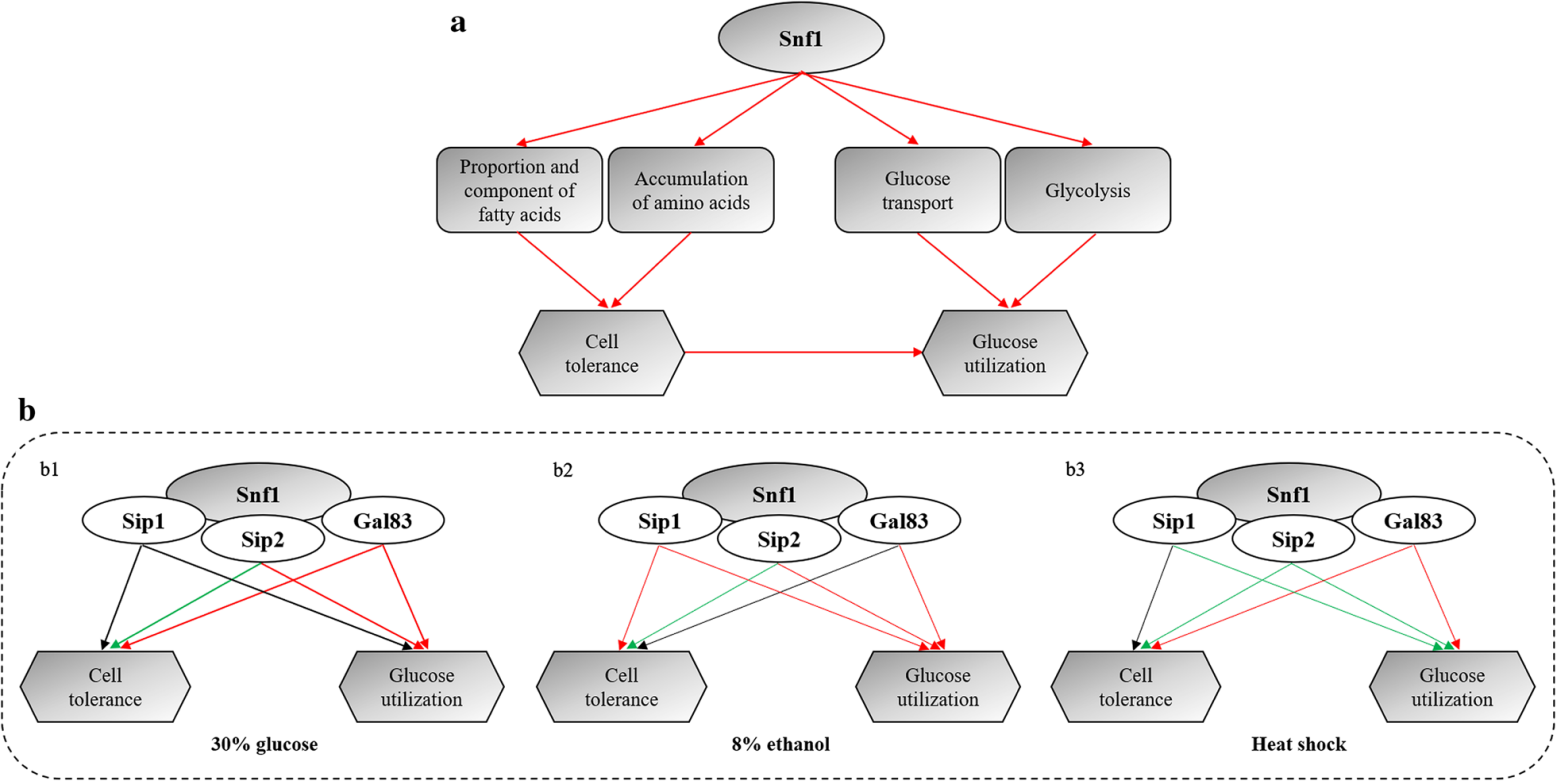
The role of Snf1 and beta regulatory subunits in enhancing multi-stress tolerance and glucose utilization. **a** the pathway of Snf1 in enhancing cell tolerance and glucose utilization in stresses. **b** the form of β regulatory subunits dominated in stresses with regard to cell tolerance and glucose utilization. Red arrow line: positive regulation. Green arrow line: negative regulation. Black arrow line: no obvious effect

The results of this study suggest that overexpression of *SNF1* is effective to improve *S. cerevisiae* cell resistance and glucose consumption in high glucose, ethanol, and heat stresses (Fig. 7), which might be related to the changed accumulation of fatty acids and amino acids and altered expression level of genes involved in glucose transport and glycolysis (Fig. 7a). However, distinguished form of β regulatory subunits dominated in stresses for cell stress and glucose utilization (Fig. 7b). The Sip1 isoform was more necessary to the growth and glucose consumption in ethanol stress. The glucose uptake largely depended on the Sip2 isoform in high sugar and ethanol stresses. The Gal83 isoform only contributed inferior effect on the growth in ethanol stress. Therefore, redundancy and synergistic effect of β subunits might occur in stresses, but each subunit showed specificity under various stresses. Although the mechanism of cell stress regulated by Snf1 needs to be further studied, this study enriches the understanding of the function of Snf1 and provides an insight to breed multi-tolerant yeast strains.

## Methods

### Strains and plasmids

The *Escherichia coli* strain DH5α and *S. cerevisiae* strain AY3α were deposited in the food biotechnology laboratory, Hainan University.

The plasmids pUG6 carrying the marker gene *KanMX* and Yep-PGK carrying the yeast phosphoglycerate kinase promoter segment *PGK1*_*P*_ and terminator segment *PGK1*_*T*_ were gifted by professor Zhang [48]. The plasmid Yep-PK carried the marker fragment. The recombinant plasmids Yep-PSK, Yep-PSI1K, Yep-PSI2K, and Yep-PG83K carried the target genes *SNF1*, *SIP1*, *SIP2*, and *GAL83*, respectively.

The *SNF1* overexpression strain α + S, *SIP1* overexpression strain α + SI1, *SIP2* overexpression strain α + SI2, and *GAL83* overexpression strain α + G83 carried the recombinant plasmids Yep-PSK, Yep-PSI1K, Yep-PSI2K, and Yep-PG83K, respectively. The blank control transformant α + K carried the plasmid Yep-PK without target genes.

### Media and growth condition

Luria–Bertani (LB) medium was used to the cultivation of *E. coli* at 37 °C, and 100 μg/mL ampicillin was supplemented to culture and screen the positive *E. coli* transformants. LB medium consists of 10 g/L tryptone, 10 g/L NaCl, and 5 g/L yeast extract.

Yeast extract peptone dextrose (YEPD) medium was used to the cultivation of *S. cerevisiae*, and 800 μg/mL G418 was supplemented to culture and screen the positive *S. cerevisiae* transformants. YEPD medium contains 20 g/L peptone, 20 g/L glucose, and 10 g/L yeast extract.

The *S. cerevisiae* cells were statically precultured in 100 mL YEPD medium in a 250 mL baffled flask at 30 °C for 24 h. Then the first-precultured cell cultures (10% inoculations) were inoculated to 200 mL YEPD medium in a 500 mL baffled flask at 30 °C for 18 h with 180 rpm rotary shaking. To investigate the effect of target genes overexpression on glucose utilization in different stresses, the second-precultured cell cultures were inoculated to 100 mL medium in a 250 mL baffled flask with the following conditions: (1) YEPD medium with 30% glucose at 30 °C, (2) YEPD medium with 8% ethanol at 30 °C, (3) YEPD medium at 42 °C. Considering the condition of actual production, 42 °C was used in the analysis of glucose utilization in heat stress.

### Construction of plasmids

Genomic DNA of *S. cerevisiae* was obtained from the parental strain AY3α using a yeast DNA kit (D1900, Solarbio, Beijing, China). Plasmids were prepared from *E. coli* DH5α using a Plasmid Mini Kit II (DC201-01, Vazyme, Jiangsu, China). The linkage of genes and plasmids was performed using a ClonExpressIIOne Step Cloning Kit (C112, Vazyme, Jiangsu, China).

The episomal plasmid Yep-PSK was constructed as follows: the *SNF1* gene was amplified by PCR from the genomes of AY3α using SNF1-F and SNF1-R primers shown in Table 1. The *SNF1* gene was cloned to the *Xho*I site of Yep-PGK, between *PGK1*_*P*_ and *PGK1*_*T*_ to generate the plasmid Yep-PS. The *KanMX* fragment was amplified by PCR from the plasmid pUG6 using Kan-F and Kan-R primers. The gene *KanMX* was inserted to the *Sph*I sites of Yep-PS and Yep-PGK to obtain the final recombinant plasmid Yep-PSK and the blank control plasmid Yep-PK, respectively.

**Table 1:** Primers used in the present study

Primer	Sequence (5′→3′)
For construction and verification	
SNF1-F	GGAATTCCAGATCTCCTCGAGATGAGCAGTAACAACAACACAAACA
SNF1-R	ATCTATCGCAGATCCCTCGAGTCAATTGCTTTGACTGTTAACGG
SIP1-F	GGAATTCCAGATCTCCTCGAGATGGGAAACAGTCCTTCTACTCAGGAT
SIP1-R	ATCTATCGCAGATCCCTCGAGCTAATTACTGATCTGAGACTTTTGTG
SIP2-F	GGAATTCCAGATCTCCTCGAGATGGGTACTACGACAAGTCATCCAG
SIP2-R	ATCTATCGCAGATCCCTCGAGTTACGAGGACTCTATGGGCGTATAAAG
GAL83-F	GGAATTCCAGATCTCCTCGAGATCTATCGCAGATCCCTCGAG
GAL83-R	ATCTATCGCAGATCCCTCGAGCTATTGCAATGGTGTATACAGTATTTGG
Kan-F	AGAGTCGACCTGCATGCCAGCTGAAGCTTCGTACGCTG
Kan-R	GCCAGTGCCAAGCTTGCATGCGCATAGGCCACTAGTGGATCTGA
PGK-F	TCTAACTGATCTATCCAAAACTGA
PGK-R	TAACGAACGCAGAATTTTC
K-F	CAGCTGAAGCTTCGTACGC
K-R	GCATAGGCCACTAGTGGATCTG
For real-time PCR	
HXT1-F	TGTGCCATTGGTGGTATCGT
HXT1-R	ACCACCGACACCTAAACCAG
HXK1-F	TACTGGTGTCAACGGTGCTT
HXK1-R	GTTCGTCGACAGCAACATCG
GLK1-F	TCTGCGACGATTTCGAGGTT
GLK1-R	CTTTGTCCGAGGCCAATGTG
PFK1-F	CAGAAAGCGTGAGGGTCGTA
PFK1-R	TCGATGGAACCGACAAGACC
PFK2-F	CGCAGTTTCAACCAAGCCAA
PFK2-R	AAGATAGCGGAACGCACGAT
TDH1-F	TTGAGGTTGTTGCTGTCAACG
TDH1-R	GCTTGTCGTCATGGGAAACAG
ACT1-F	ACGCTCCTCGTGCTGTCTTC
ACT1-R	GTTCTTCTGGGGCAACTCTCA

The episomal plasmids Yep-PSI1K, Yep-PSI2K, and Yep-PG83K were constructed as Yep-PSK using primers SIP1-F/SIP1-R, SIP2-F/SIP2-R, and GAL83-F/GAL83-R, respectively.

### Transformation of yeast

The plasmids Yep-PSK, Yep-PSI1K, Yep-PSI2K, Yep-PG83K, and Yep-PK were transferred to the parental strain AY3α according to the previous study [49]. The transformants α + S, α + SI1, α + SI2, α + G83, and α + K were verified by PCR using the primers PGK-F/PGK-R and K-F/K-R shown in Table 1.

### Analysis of growth property

The strains were precultured in YEPD medium at 30 °C overnight. The cell cultures were adjusted to OD600 = 1.2, and 4% inoculations were transferred to the YEPD medium to determine the growth curve at 30 °C. The cell density (OD600) was detected with a UV spectrophotometer (T6, Persee, Beijing, China).

### Analysis of stress tolerance

The second-precultured cell cultures were cultivated in YEPD medium to logarithmic phase and were adjusted to OD600 = 1.0. The cell suspension was treated in YEPD medium with 30% glucose at 30 °C for 30 min, treated in YEPD medium with 8% ethanol at 30 °C for 30 min, and treated in YEPD medium at 53 °C for 2 min, respectively. To better reflect the effect of Snf1 on the growth of yeast cells in heat stress, 53 °C was used in the analysis of heat stress tolerance.

The cell cultures (1 mL) were sampled and diluted to an appropriate concentration. After culturing in the YEPD plates at 30 °C for 2 days, the relative cell viability was calculated and expressed as the relative percentage of colony forming units (CFUs) after stressing to the CFUs before stressing.

### Analysis of sugar consumption

For the measurement of extracellular glucose, cultures were sampled at suitable intervals. Samples were analyzed using high-performance liquid chromatography (HPLC) as previously [49]. The glucose utilization efficiency was determined by the ratio of the consumed glucose in total sampling time with the total glucose.

### Analysis of fatty acids (FAs)

After stresses treated, the cells (dry weight 5.4 mg) were harvested to the determination of fatty acids and amino acids.

The cells were mixed with 1 mL of sodium hydroxide methanol solution (45 g NaOH, 150 mL distilled water, and 150 mL methanol) at 80 °C in a water bath with intermittently stirring. After saponification for 30 min, 2 mL of 6 M methanol hydrochloride solution mixed at 80 °C for 30 min. The fatty acid methyl esters were extracted with 2 mL hexane and washed with 2 mL distilled water for analysis.

The fatty acids were determined by gas chromatography–mass spectrometry using an Agilent 7890 GC (Agilent Technologies Inc., Santa Clara, CA, USA) with an Agilent 5975 mass spectrometer system. The separation was carried out in a HP-5MS column (60 m × 0.25 mm inner diameter, 0.25 μm film thickness, Agilent Technologies Inc.) with split injection (20:1). The carrier gas Helium kept at 1.5 mL/min. The initial temperature of the column was 120 °C for 1 min, firstly raised to 170 °C at a rate of 6 °C/min, secondly raised to 215 °C at a rate of 2.5 °C/min for 12 min, thirdly raised to 230 °C at a rate of 4 °C/min for 10 min, and finally raised to 280 °C at a rate of 10 °C/min for 15 min. The injection volume was 1.0 μL. The temperatures of the injection port, ion source, four-stage bar, and connecting wire were 280 °C, 200 °C, 150 °C, and 260 °C, respectively. The mass spectra gained in an electron impact mode of 70 eV, and the mass (m/z) range scanned from 40 to 550 atomic mass units. The fatty acids were identified by retrieving NIST11 Database (Agilent Technologies), referring to the chromatogram and retention time of the standards. The content of fatty acids was expressed as the relative percentage of identified fatty acids.

### Analysis of amino acids

The cells were mixed with 2 mL of 6 M HCl (1% phenol added), and nitrogen was filled for 1 min. After hydrolyzing at 110 °C for 22 h, the mixture was supplemented to 2 mL and dried by nitrogen. After dissolving in 0.01 M HCl, the samples were filtered.

The amino acids were derivatized using an automatic on-line derivatization method of Agilent. The primary and secondary amino acids were reacted with phthalaldehyde (OPA) and fluorene methoxycarbonyl chloride (FMOC), respectively.

HPLC was used to determine the amino acids with an Agilent 1100 apparatus and a ZORBAX Eclipse AAA column (4.6 × 150 mm, 3.5 μm). Sodium dihydrogen phosphate (40 mM, pH7.8) was used as the mobile phase A. The mobile phase B contained acetonitrile, methanol, and water (volume ratio 45:45:10). The gradient was 0% B (0 min), 0% B (1.9 min), 57% B (18 min), 100% B (18.6 min), 100% B (23 min), 0% B (23.2 min), and 0% B (27 min). The fluorescence detection (EX = 266 nm, EM = 305 nm) was used to the test of proline. The other amino acids were measured at 338 nm in ultraviolet detection. The amino acid standard (AAS-18, Sigma, Saint Louis, MO, USA) was used in the identification and quantification, and the results were expressed in mg/g cell dry weight (CDW).

### Real-time quantitative PCR (RT-qPCR)

The cells sampled before and after stresses, and the expression levels of genes related to glucose transport and glycolysis were tested. The RT‑qPCR was conducted as Zhang et al. [48]. The primers for the target genes *HXT1* encoding hexose transporter, *HXK1* encoding hexokinase 1, *GLK1* encoding glucokinase, *PFK1* encoding 6-phosphofructokinase subunit alpha, *PFK2* encoding 6-phosphofructokinase subunit beta, *TDH1* encoding glyceraldehyde-3-phosphate dehydrogenase, and the reference gene *ACT1* are shown in Table 1.

### Statistical analysis

Results are average data from at least three experiments, and error bars represent the standard deviations. Student’s *t* test was conducted to confirm the differences between the transformants and the wild type. The differences of *P* < 0.05 were considered statistically significant.

## Supplementary information

**Additional file 1.** Analysis of the relative cell viability of the strains in stresses. The cells were cultivated in (a) 70 g/L glucose at 30 °C, (b) 300 g/L glucose at 30 °C, (c) YEPD medium with 8% ethanol at 30 °C, and (d) YEPD medium at 53 °C. The relative cell viability was calculated and expressed as the relative percentage of colony forming units (CFUs) after stressing to the CFUs before stressing. Significant difference of the transformants from the parent strain AY3a was confirmed by Student’s t-test (*P < 0.05).

## Data Availability

All data generated or analysed during this study are included in this published article.
