# Supplementary material for: Enhanced multi-stress tolerance and glucose utilization of Saccharomyces cerevisiae by overexpression of the SNF1 gene and varied beta isoform of Snf1 dominates in stresses
Source: Microb Cell Fact. 2020 Jun 22;19:134. doi: 10.1186/s12934-020-01391-4 (PMC7310068; doi:10.1186/s12934-020-01391-4)
Supplement: Supplementary file 1 — Additional file 1. Analysis of the relative cell viability of the strains in stresses. The cells were cultivated in (a) 70 g/L glucose at 30 °C, (b) 300 g/L glucose at 30 °C, (c) YEPD medium with 8% ethanol at 30 °C, and (d) YEPD medium at 53 °C. The relative cell viability was calculated and expressed as the relative percentage of colony forming units (CFUs) after stressing to the CFUs before stressing. Significant difference of the transformants from the parent strain AY3a was confirmed by Student’s t-test (*P < 0.05). [file 12934_2020_1391_MOESM1_ESM.docx]

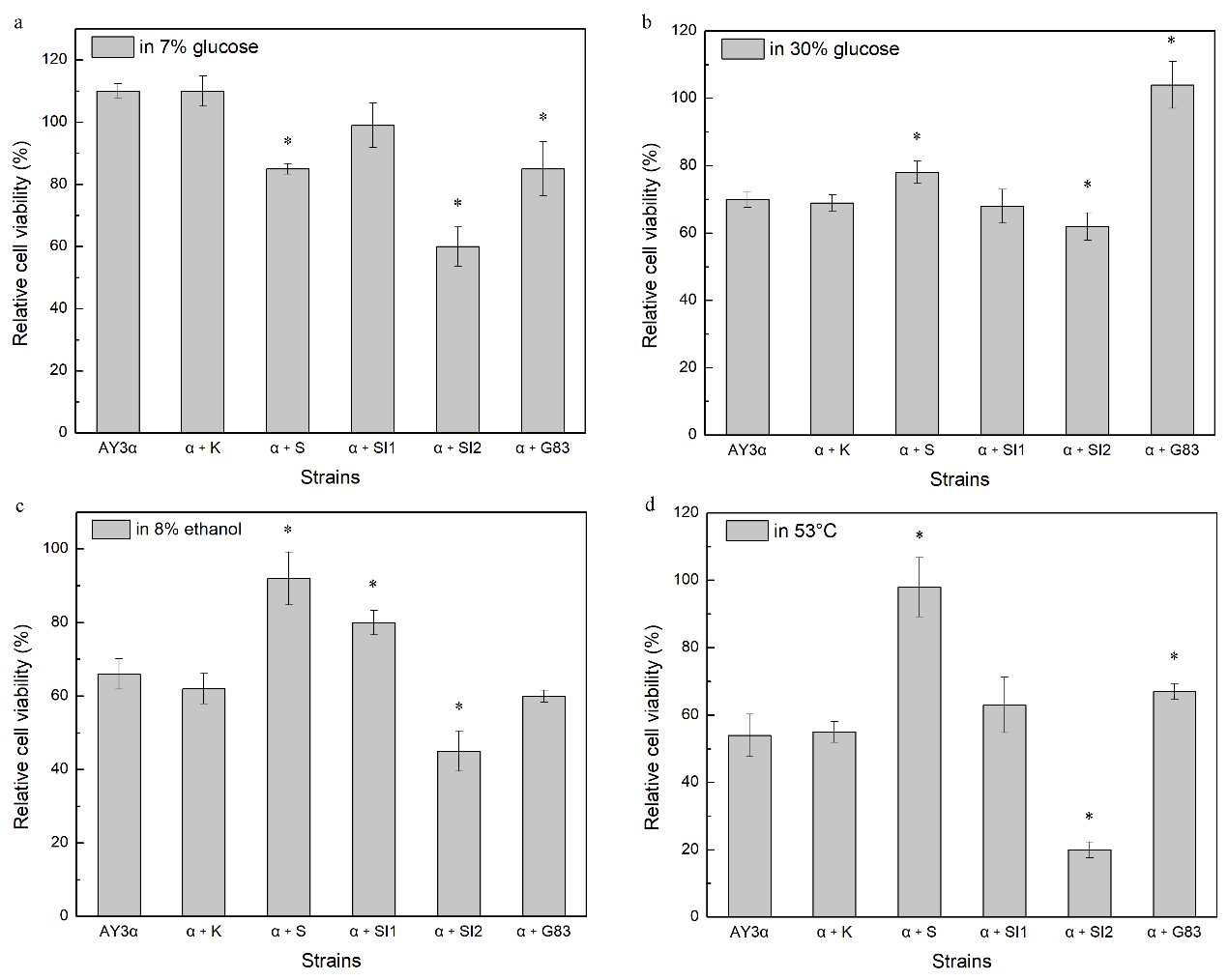


Analysis of the relative cell viability of the strains in stresses.

The cells were cultivated in (a) 70 g/L glucose at 30°C, (b) 300 g/L glucose at 30°C, (c) YEPD medium with 8% ethanol at 30°C, and (d) YEPD medium at 53°C. The relative cell viability was calculated and expressed as the relative percentage of colony forming units (CFUs) after stressing to the CFUs before stressing. Significant difference of the transformants from the parent strain AY3a was confirmed by Student’s t-test (^*^*P* < 0.05).
